# Cross-Sectional Analysis of the Challenges Faced by Undergraduate Dental Students During Root Canal Treatment (RCT) and the Oral Health-Related Quality of Life in Patients After RCT

**DOI:** 10.3390/medicina61020215

**Published:** 2025-01-25

**Authors:** Mubashir Baig Mirza, Abdullah Bajran Almuteb, Abdulaziz Tariq Alsheddi, Qamar Hashem, Mohammed Ali Abuelqomsan, Ahmed AlMokhatieb, Shahad AlBader, Abdullah AlShehri

**Affiliations:** College of Dentistry, Prince Sattam Bin Abdulaziz University, Al-Kharj 11942, Saudi Arabia; 438050906@std.psau.edu.sa (A.B.A.); 441051147@std.psau.edu.sa (A.T.A.); q.hashem@psau.edu.sa (Q.H.); m.abuelqomsan@psau.edu.sa (M.A.A.); a.almokhatieb@psau.edu.sa (A.A.); s.albader@psau.edu.sa (S.A.)

**Keywords:** dental students, oral health, patient outcomes, quality of life, root canal therapy

## Abstract

*Background and Objectives*: This study examined dental students’ challenges with root canal treatment (RCT). It also assessed patients’ perceptions of oral health-related quality of life (OHRQoL). *Materials and Methods*: The study utilized three prevalidated questionnaires. One questionnaire was administered to dental students to assess their challenges related to various aspects of RCT. Another questionnaire was distributed to patients, comparing demographic factors such as age and gender to the tooth type, pain scores before and after RCT, and socioeconomic status. The third questionnaire focused on patients’ OHRQoL considering age, gender, tooth types, pain, and socioeconomic status using the Oral Health Impact Profile-14 scale (OHIP-14). A total of 75 dental students filled out the survey, and patient-related questionnaires were filled out by 585 patients with the following demographics: age: young adults n = 385 (65.81%), middle-aged adults n = 200 (34.19%); gender: males n = 366 (62.56%), and females n = 219 (37.44%). Categorical data were analyzed using frequency and percentage. Chi-square tests were used for comparative analysis, and one-way ANOVA was used when more than two variables were present. A *p*-value of ≤0.05 was set as statistically significant. *Results*: Dental students perceived themselves as competent in performing RCT but faced difficulties with canal localization in middle-aged patients (*p* < 0.01) and in communicating with female patients (*p* = 0.009). There was a significant difference in preoperative (*p* = 0.007) and postoperative pain levels (*p* = 0.003) when comparing genders. Overall, there was a 30.60% reduction in pain levels. The OHIP-14 scale indicated high patient satisfaction (78.9%), with physical disability (26.16%) and psychological discomfort (23.33%) being the most affected domains. Among different variables, socioeconomic status was statistically significant, with patients of lower socioeconomic status reporting higher satisfaction levels (*p* = 0.02). No significant differences in OHRQoL were found based on age or gender. *Conclusions*: The OHRQoL was high among patients treated by dental students, with those with a low socioeconomic status being the most satisfied.

## 1. Introduction

Root canal treatment (RCT) plays a vital role in preserving teeth impacted by pulp diseases, with its long-term success heavily dependent on the quality of the procedure [1]. It is one of the most frequently performed dental procedures worldwide. In Saudi Arabia, the percentage of patients receiving RCT is notably high, primarily due to the increased incidence of dental caries in both primary and permanent teeth [2]. As of 2022, specialist endodontists make up only 3.4% of the dental workforce, which increasingly consists of general dentists due to the continuous influx of new graduates each year [3]. Research indicates that RCT carried out by specialists yields substantially improved outcomes, enhanced healing, and better patient acceptance [4]. However, due to increasing demand and limited specialists, general dentists often perform RCT. Dental schools must understand the obstacles students face and their challenges while performing endodontic treatment, as this understanding is critical to providing students with sufficient clinical exposure and training [5].

The national population index of Saudi Arabia indicates that a substantial portion comprises young adults with a median age of 29 years [6]. Understanding the differences in endodontic treatment outcomes among different age groups is crucial for public health planning. Greater oral health awareness has increased dental care demand among older adults [7]. Although the principles of endodontic treatment are similar, dentists encounter unique challenges in middle-aged adults due to age-related changes in pulp tissues, though these changes are not as pronounced as in the elderly [8]. Research suggests that treating patients over 40 is challenging due to medical conditions, unfavorable prognosis, and technical difficulties. Additionally, age-related changes in tooth morphology, such as obliterated root canals, can further complicate treatment [9].

Some studies suggest that age may not significantly impact the outcomes of RCT. In contrast, others indicate that older patients might experience lower success rates and a greater likelihood of requiring post-treatment extractions, as they often have a higher prevalence of apical periodontitis [10,11]. Tooth-specific factors, such as missed canals and inadequate root canal filling, are significant risk indicators for periapical pathosis in teeth that have undergone RCT [7,11]. Research has also shown notable associations between periapical pathosis and various factors, including gender, smoking status, education level, and socioeconomic status [12,13]. However, many of these studies have yet to distinguish between the periapical pathosis observed in root-filled versus untreated teeth. 

Many studies have investigated the clinical aspects of endodontic treatment, but there remains a considerable gap in research regarding treatment outcomes and quality of life across various demographic and clinical factors [8,13,14]. The methods used to evaluate healthcare quality have evolved significantly over time. Initially, assessments focused primarily on conventional methods emphasizing clinical procedures and outcomes. However, a distinct shift has emerged toward a more patient-centered model of care [15,16]. This modern perspective emphasizes the importance of patients’ experiences, preferences, and needs, ensuring that the quality of treatment is evaluated not only by clinical results but also by the satisfaction and engagement of those receiving endodontic care. The dental community must recognize and prioritize patient satisfaction in their practice, as it significantly impacts the overall success of the treatment [16].

Endodontic procedures enhance the oral health-related quality of life (OHRQoL) [17]. The outcomes and satisfaction of patients are vital for evaluating the success of treatment. One way to gauge this is by employing the Oral Health Impact Profile (OHIP-14) scale to assess patient satisfaction. The original OHIP, developed based on Locker’s conceptual model and the World Health Organization’s International Classification of Impairments, Disabilities, and Handicaps, consisted of 49 items [13]. This tool has since been restructured into a shorter version, known as the OHIP-14, which contains 14 items that span seven key domains: functional limitations, physical pain, psychological discomfort, physical disability, psychological difficulty, social disability, and handicap. The OHIP-14 offers valuable insights into patients’ perceptions of endodontic disease and treatment [8].

This study primarily aims to compare the patient treatment factors and challenges dental students encounter when performing RCT on middle-aged versus younger patients and between genders. Additionally, the study aims to evaluate the OHRQoL of patients who received RCT from dental students at the dental school’s endodontic facility.

## 2. Materials and Methods

This study employed a retrospective cross-sectional design utilizing questionnaire-based surveys. The sample size was calculated using the RaoSoft sample size calculator (Raosoft Inc., Seattle, WA, USA), determining a target of 500 patients and 70 operators, with an absolute error of 5% and a confidence interval of 95%. 

Participant Recruitment: Data collection occurred from February 2022 to October 2024, encapsulating two years, with operational timelines extending from February 2022 to October 2024. A total of 660 participants were recruited, comprising 585 patients who had undergone RCT and 75 dental students from the College of Dentistry at Prince Sattam bin Abdulaziz University (PSAU). Two primary recruitment methods were employed: 1. Direct Recruitment in Clinics: Patients seeking dental care who had received RCT from dental students within the past two years were approached in the clinics where they sought additional dental care, either for endodontic (follow-up visits or new RCTs) or other specialty treatments. Information about the study was disseminated through flyers containing barcodes with survey links, facilitating participation. Dental students obtained informed consent from interested patients and conducted direct survey recruitment. This method garnered 396 responses over seven months (February to August 2024). 2. Email Recruitment from Central Database: The email addresses of patients who had undergone RCT at the clinics within the last two years were retrieved from the institution’s central database. An initial email detailing the study containing survey links and consent forms was sent to 412 patients. Due to undeliverable email addresses, only 399 patients were successfully contacted; 144 responses were initially received after one month, followed by an additional 45 responses after a reminder email was sent to non-respondents.

Dental Student Participation: Dental students who participated in the clinical endodontics and comprehensive clinical dentistry programs during the study period were invited to complete a questionnaire via email. Within 14 days, 59 responses were recorded, with a subsequent reminder email resulting in an additional 28 responses. Twelve student surveys were excluded from the analysis due to incomplete data.

This study received ethical approval from the standing committee for bioethics research at PSAU, with approval number SCBR-224/2024.

### 2.1. Inclusion and Exclusion Criteria

All patients must have undergone RCT on at least one tooth conducted by students within the dental school in the last two years and must be at least 18 years of age. Furthermore, at least one week must have elapsed since the completion of RCT before completing the questionnaire. The study imposed no restrictions related to gender. Participants who declined to engage, did not provide informed consent, or submitted incomplete questionnaires were excluded from the research.

### 2.2. Questionnaires

The present study comprises three questionnaires: two administered to patients and one directed towards the operators.

### 2.3. Operator-Based Instrument

The prevalidated questionnaire used for dental students aimed to assess their difficulties in performing RCT on different patient groups [8]. The questions related to communication difficulties, diagnostic procedures, and technical aspects of RCT, encompassing access cavity preparation, rubber dam application, localization of root canal, working length determination, instrumentation, and obturation procedures. Operators were asked to rate each question using a 3-point Likert scale: “easy”, “average”, or “difficult”.

### 2.4. Patient-Based Instrument

Two questionnaires assessed patient-reported outcomes and OHRQoL. The first questionnaire was based on previously validated survey analyses by Petrauskiene and Haug [8]. It focused on the experience of pain before and after RCT, with questions categorized by age and gender. It was a close-ended questionnaire, where the pain was rated on a scale of 1 to 2 (score 1: yes; score 2: no). Patients were also asked about the teeth that underwent RCT and their socioeconomic status on a scale of 1 to 3 (1: high-income group; 2: middle-income group; 3, low-income group).

The second questionnaire was about OHRQoL, using the OHIP-14 survey. The previously validated Arabic version of the OHIP-14 was used [14]. The patients filled out the OHIP-14 form individually. A 5-point Likert scale was used to answer each question of the OHIP-14 scale, which contained variables like functional limitations, pain, discomfort, and disabilities (psychological and physical). Each question was evaluated using the following response scale: 0 for “never”, 1 for “hardly ever”, 2 for “occasionally”, 3 for “fairly often”, and 4 for “very often”. The individual domain score was calculated by summing the responses of two items within a specific domain. The total OHIP-14 summary score was obtained by adding the responses to all items. Higher scores indicated a lower OHRQoL. The Cronbach alpha coefficient for all 14 items in the OHIP-14 questionnaire was established to be 0.897, which is considered good.

### 2.5. Statistical Analysis

All data were represented as frequency and percentage (for categorical variables). The chi-square test was employed to analyze the association between two categorical variables, while one-way ANOVA was utilized to assess differences among means across multiple groups. Fisher’s exact test was used for comparative analysis when observed frequencies were less than five. A calculated P-value less than 0.05 was considered statistically significant. All the analyses were conducted using the commercially available statistical package SPSS v.23 for Windows (IBM Corp, Armonk, NY, USA).

## 3. Results

A total of 585 patients and 75 undergraduate dental students participated in this questionnaire-based study. Most patients were young adults (between 20 and 39 years of age; n = 385; 65.81%), while the remaining were middle-aged adults (between 40 and 59 years of age; n = 200; 34.18%). Most participants were male (n = 366; 62.56%) compared to female (n = 219; 37.44%).

Table 1 compares the challenges faced by dental students during RCT on patients of different age groups and genders. The chi-square test of association revealed no significant relationship between age and the various levels of communication, diagnosis, rubber dam application, access cavity preparation, working length establishment, instrumentation, and obturation (*p*-value > 0.05). The only exception was canal localization, demonstrating a significant association (*p*-value < 0.01). This finding suggests that students found canal localization more challenging in older than younger patients. Similarly, gender is not significantly associated with any of the difficulties surveyed (*p*-value > 0.05), except while communicating with the patients, with students finding it more challenging to communicate with female patients effectively (*p*-value < 0.009).

Table 2 displays the OHIP-14 data with mean and standard deviations related to the seven domains. These data reveal the challenges experienced by patients following endodontic treatment, with prevalence rates ranging from 13.08% to 26.16%.

The most frequently reported issue was physical difficulty, affecting 26.16% of patients, followed by psychological difficulty at 23.33%. Additional concerns included social disability (22.31%), psychological discomfort (21.8%), physical pain (21.45%), functional limitation (19.58%), and handicap (13.08%). Regarding individual queries, the patients felt the most difficulty relaxing after RCT, 32.5%, and the least related to dissatisfaction with lifestyle after RCT, 11.6%.

Table 3 reveals a significant association for both genders concerning pain experienced both before (*p*-value 0.007) and after RCT (*p*-value 0.003), indicating that male patients are more likely to report pain. However, no significant differences were found in RCT outcomes based on the type of tooth treated or the patients’ socioeconomic status. When considering age groups, the data show a significant association regarding preoperative pain, with younger patients more frequently seeking treatment due to pain compared to the middle-aged group (*p*-value < 0.01). No statistical differences were observed among other factors in relation to the age of patients.

As shown in Table 4, similar total OHIP values were observed when comparing different age groups, with no statistical differences in any of the domains (*p*-value 0.500). The only exception was functional limitations, where younger patients demonstrated a better OHRQoL following endodontic treatment (*p*-value 0.018).

Regarding gender, female patients’ total OHIP mean values were slightly lower (3.731) than male patients (4.019), indicating a slightly better OHRQoL among females. However, no significant differences were found across domains based on patient gender, suggesting that the OHIP is consistent across different genders (*p*-value 0.541 ns).

**Figure 1:**
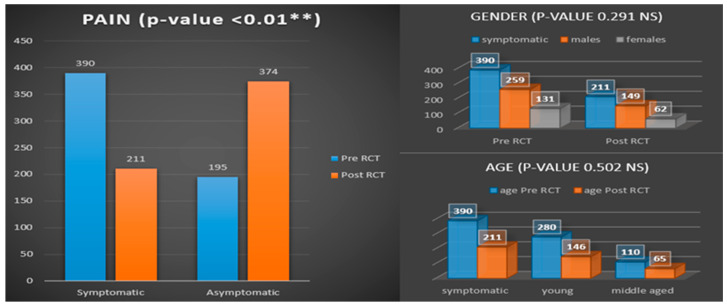
Illustration of pain before and after RCT. Symptomatic, presence of pain; asymptomatic, absence of pain; NS, nonsignificant difference with *p*-value ≤ 0.05; RCT, root canal treatment. ** *p* < 0.01.

Regarding pain, the OHRQoL based on the mean values pertaining to specific domains associated with pain, both before and after RCT, indicated no statistically significant difference, as evidenced by *p*-values of 0.966 and 0.310, respectively. However, using the data from Table 2, as illustrated in Figure 1, a 30.06% decrease in post-RCT pain was seen compared to the number of patients presenting with preoperative pain.

Patients with low socioeconomic status reported higher satisfaction, as indicated by their lower mean values in the various domains. Significant differences were observed across all domains based on the patient’s socioeconomic status, except for physical pain (*p*-value = 0.731) and handicap (*p*-value = 0.208). When comparing OHIP based on RCT for different types of teeth, significant differences were seen in physical pain (*p*-value 0.044) and physical disability (*p*-value 0.021). Specifically, anterior teeth exhibited the lowest mean values in all categories, indicating patients were more satisfied after RCT in anterior teeth. In queries related to psychological difficulties, patients with RCT in the anterior and molars reported similar values to those with RCT on anterior teeth only. However, when considering the total OHIP score across all domains for different teeth, the differences were not statistically significant, with a *p*-value of 0.085.

## 4. Discussion

The consensus-based checklist for reporting survey studies (CROSS) was applied for reporting these surveys. The findings of this study did not indicate significant differences in the technical aspects of RCT among various age groups and genders. However, dental students reported facing more significant challenges when locating root canals in middle-aged patients than younger ones. Additionally, a statistically significant difference was only observed in communication with patients, with dental students facing challenges in interactions with female patients. Regarding OHRQoL, the overall satisfaction was high after RCT (78.9%) with patients belonging to low socioeconomic status being the most satisfied. The patients were least satisfied with physical difficulty.

### 4.1. Demographic and Patient-Related Factors

#### 4.1.1. Age

In this study, dental students faced difficulty locating canals, especially in older patients. Similar challenges have been reported in previous research involving dental students in Brazil and Turkey [18,19]. The tooth’s location compounds the challenge of canal location; posterior teeth typically have more canals, some of which may be obscured by dentin, making them harder to identify even for experienced dentists [20]. The advanced age of patients often exacerbates these challenges, increasing the likelihood of pulp chamber and root canal calcification. This finding is consistent with previous research highlighting the difficulties of conducting RCT in older populations [8]. Using a dental operating microscope has been shown to alleviate some of these difficulties; however, it poses challenges in an undergraduate dental setting due to the substantial financial investment and the steep learning curve required to master the technique. As an alternative, dental loupes can be a practical solution for undergraduate students, as reported in other studies [21]. Currently, while using loupes is encouraged among students at the dental school in PSAU, it is not mandatory.

#### 4.1.2. Gender

Dental students in this study faced communication challenges related to gender, rather than difficulties in the technical aspects of performing RCT. Dentists’ communication skills are crucial for better patient health outcomes [22]. Women tend to experience more dental anxiety than men, which could be a possible reason for confusion and failure to communicate effectively [23]. On the other hand, dental students often underestimate their communication abilities due to a lack of confidence, which was previously noted to improve with more experience [24]. To combat such issues, dental schools in some countries are trying to emphasize communication skills through competency assessments, recognizing their importance in clinical practice [25]. 

In medical practice, it is well documented that patients often exhibit a preference for same-sex practitioners, primarily due to the sensitive nature of certain health issues. However, a sex-concordance effect in patient preferences for dentists in the literature reveals a more nuanced perspective [26]. It has been observed that patients frequently prefer same-sex dentists, as this can mitigate feelings of embarrassment and apprehension associated with physical examinations [27]. Additionally, some patients demonstrate a slight preference for female dentists, who are often perceived to possess greater compassion and empathy. Female practitioners tend to invest more time in discussing health concerns, which can enhance patient comfort during interactions [28]. In the context of our study, while these dynamics were not directly assessed, it is prudent to consider their potential impact on communication, particularly given that all dental students at PSAU are male. 

#### 4.1.3. Tooth Type

This study identified molars as the teeth most frequently subjected to RCT, followed by premolars. Their early eruption and complex occlusal morphology render them particularly vulnerable to dental caries, contributing to a higher incidence of endodontic intervention [20]. However, the study’s results revealed no significant differences in the frequency of RCT for molars compared to other teeth, regardless of age or gender. Similar results were also seen in other observational studies researched among dentists worldwide [29]. Previously, contrastingly, higher demand for RCT was noted among female and younger patients, while in older individuals, more anterior teeth required RCT [30].

#### 4.1.4. Pain

In this study, a statistically significant number of patients reported experiencing preoperative pain, which is recognized as the primary reason patients seek endodontic treatment. Other studies have also shown that many patients seeking RCT reported preoperative pain, consistent with our findings [31,32,33]. In contrast, a study in Brazil reported that more than 42.5% of the patients at the first dental appointment were asymptomatic [34]. Compared to older adults and males, a higher prevalence of symptoms among young adults and females has often been reported in patients referred for endodontic treatment [33]. However, in the present study, male patients demonstrated statistically significant differences in pain levels compared to females, particularly among young adults. Conversely, a Swedish study presented findings that contradicted these observations, indicating a greater prevalence of pain in older individuals without any discernible gender predilection [35].

Postoperative pain can be a complication of RCT, which can be influenced by various factors, including preoperative status, treatment techniques, time of evaluation, and clinician experience [36,37]. Although frequently encountered immediately postoperative, it dramatically declines within a week to as low as 11% [13,36]. There is no age-related difference in the occurrence or severity of pain in this study, which is in accordance with that reported in the Norwegian population [8]. Previous studies have identified more postoperative pain in females, which is in contrast to the results in this research, where males had statistically significant pain [15]. Comparing the preoperative pain to pain postoperatively, a 30.06% reduction in symptoms was noticed. However, no statistically significant difference was observed between age and gender. 

#### 4.1.5. Socioeconomic Status

This study found no differences in patient preferences for RCT based on socioeconomic factors related to gender and age, suggesting a uniform inclination towards RCT across these demographics. In contrast, prior research has indicated that individuals from lower socioeconomic backgrounds often prefer tooth extraction over RCT and demonstrate significantly poorer oral health [15]. However, a recent study showed no statistically significant difference between age groups comparing the RCT based on socioeconomic status [37].

### 4.2. OHRQoL

Patient feedback surveys and a patient-centered approach in endodontics are essential for enhancing overall patient satisfaction. Numerous studies have examined the impacts of RCT on the quality of life, employing shortened versions of the OHIP [8,13,15]. The findings from these investigations frequently indicate that RCT significantly improves OHRQoL. In this study, the assessment of OHRQoL following RCT, utilizing the OHIP-14 scale, yielded favorable results. Specifically, 78.9% of patients reported a satisfactory experience, while 21.1% reported experiencing problems occasionally. Notably, none of the patients indicated that they encountered these issues on a continual basis. The results were similar to those reported from a study at Jordan University, where over 90% of subjects stated that their quality of life improved after RCT. Interestingly, no difference was found in their study in OHRQoL after RCT was performed by specialists or undergraduate students [14].

Physical disability, along with psychological difficulties, constituted the primary domains in which patients exhibited elevated OHIP scores, indicative of diminished OHRQoL. Among these domains, the most substantial barrier to OHRQoL reported by patients pertained to their challenge in achieving relaxation following RCT. On the other hand, the patients responded with lower scores for queries related to the handicap domain, which included questions related to inability to live and dissatisfaction with lifestyle after receiving RCT, indicating a better OHRQoL. Other studies have reported that physical pain and psychological discomfort were the most affected domains after RCT [15,38].

#### 4.2.1. OHRQoL Related to Age

OHRQoL is influenced by age, with some studies indicating that elderly patients generally express greater satisfaction than younger patients, while other studies suggest the contrary [8,15]. This study’s overall OHI score did not show statistically significant differences between the young and middle-aged groups. Notably, the only domain that showed a significant difference compared to the younger age group was functional limitation, with middle-aged individuals expressing dissatisfaction regarding the deterioration of taste and difficulties in speaking following RCT. The elderly were not included in this study due to a negligible number of patients reporting for treatment in the dental school who were over 60 years of age. Several factors might explain this trend, such as the longer waiting periods in dental colleges compared to private clinics, as well as the anxiety associated with being treated by dental students [14]. Additionally, as previously mentioned, the overall population of individuals in that age group is particularly sparse in the Kingdom.

#### 4.2.2. OHRQoL Related to Gender

Regarding gender in this study, both males and females had similar responses with no statistically significant difference in the total OHIP score or individual domains. These results are in accordance with the results seen in some other studies [39]. However, most studies have shown that the OHRQoL after RCT was poorer among female patients than males. This trend was usually attributed to the consciousness about health and appearance more in females than males [8].

#### 4.2.3. OHRQoL Related to Pain

Assessing preoperative and postoperative pain is essential for enhancing OHRQoL among patients. Research conducted by Liu et al. has established that preoperative pulpal and periapical diagnoses significantly influence OHRQoL, with patients experiencing preoperative pain reporting a deterioration in their quality of life [38]. Furthermore, a study carried out in Norway identified preoperative pain as a major adverse factor affecting OHRQoL [8]. In this study, while the prevalence of preoperative pain was recorded at 66.66%, it did not demonstrate a significant impact across various domains of the OHIP-14 questionnaire.

Persistent pain has been shown to negatively affect a patient’s perceived OHRQoL. Contributing factors include unsuccessful treatment outcomes, iatrogenic procedures, and tooth loss [38]. In our study, many patients reported experiencing pain relief following RCT. Despite the lack of a standardized timeframe for evaluating pain, this study focused on patients who underwent RCT within the last two years, ensuring that none had received the procedure less than one week before participating, which aligns with a similar study conducted in Spain [15]. A previous study in Canada excluded participants who had visited the dentist within the prior three months to mitigate bias related to recent treatment experiences, underscoring the variability in methodologies concerning pain assessment [13].

#### 4.2.4. OHRQoL Associated with RCT on Various Tooth Types

Prior research has identified tooth types and their positions within the dental arch as significant determinants that influence the quality of treatment outcomes [40]. In this study, only physical pain and physical disability showed statistically significant differences, while no distinctions were found between the types of teeth treated across the other five domains. Additionally, the overall OHIP score did not indicate any statistically significant differences between tooth types; however, patients reported greater satisfaction regarding the treatment outcomes for anterior teeth. It is plausible that the RCT performed on anterior teeth was influenced by aesthetic considerations, which may have been addressed following the restoration of these teeth post-RCT, as indicated in previous research [8,15]. Furthermore, the challenges associated with RCT in posterior teeth, coupled with the inherent risks of complications when performed by dental students, may account for the observed variations in the domains of physical pain and disability.

#### 4.2.5. OHRQoL Related to Socioeconomic Status

Patients with low socioeconomic status reported significantly improved OHRQoL across all domains, as evidenced by a lower overall OHIP score and reduced mean scores in each domain. In contrast, middle-income patients expressed the lowest levels of satisfaction, reflected in the highest overall OHIP scores, particularly concerning functional limitations, physical pain, social disability, and handicap. High-income patients also reported lower satisfaction levels, with elevated mean OHIP scores related to psychological discomfort, psychological difficulties, and physical disability. In contrast to our study, Montero et al. reported that participants from higher socioeconomic backgrounds experienced greater OHRQoL following RCT [15]. Their study involved all RCT procedures conducted by a single experienced dentist, whereas our research included multiple dental students performing the treatments. Numerous studies have consistently reached analogous conclusions based on their findings, particularly concerning the QoL of economically disadvantaged patients [41].

Interestingly, studies conducted in Brazil align with our findings, revealing that post-treatment satisfaction levels are notably higher among patients from lower socioeconomic backgrounds regarding a range of dental care services [42,43]. Although such outcomes are infrequent, they can be anticipated for several reasons. Research indicates that the financial burden associated with RCT is the most common deterrent hindering patient satisfaction [44,45]. Given that the dental services provided at PSAU clinics are offered at no cost and are fundamentally designed for the community’s welfare, this model was the most likely reason to enhance patient satisfaction, particularly among low-income individuals. The role of a sense of coherence in QoL has also been recently investigated [46]. It refers to an individual’s capacity to navigate stressful situations and overall life perspective. Zakershahrak et al. found that patients with a robust sense of coherence—despite belonging to a low socioeconomic status—exhibit improved QoL [46]. Although assumptive, its role should further be evaluated in the context of the results from our study. 

This study design has some limitations due to the retrospective nature of our study. Maintaining a uniform evaluation timeframe proved challenging; thus, after reviewing the literature, we established a two-year timeline for several reasons, some of which are supported by previous longitudinal studies [44,47,48]. The following conclusions were drawn from these studies, which influenced the timeline in this study: Following RCT, a two-year recall period enables the resolution of transient symptoms, thereby ensuring that the OHRQoL assessment accurately reflects the actual treatment outcomes rather than temporary post-surgical effects. However, we acknowledge that recall bias may have influenced some of the responses. Considering the social and psychological dimensions of patient well-being, we determined that a more extended evaluation period was essential, as these aspects may evolve as individuals integrate treatment outcomes into their overall quality of life. Conversely, symptoms such as pain and discomfort are expected to diminish within a few days. Therefore, as emphasized in the manuscript, a follow-up period of less than seven days was intentionally not employed. Furthermore, a shorter interval may not adequately capture the treatment’s effectiveness. The variability in patient assessment timeframes could partially account for differences in their responses. This observation is emphasized by the findings in this study, where physical pain was notably lower than physical discomfort and psychological disability. 

The technical quality of RCT could play a pivotal role in long-term success. However, this study did not evaluate it because of the research’s cross-sectional design, which is inherently limited in its capacity to appraise the technical quality. It was aimed at self-reported responses garnered from patient responses to find their satisfaction levels and the influence of any demographic factors. While the study provides valuable insights into the challenges and outcomes of RCT in different age groups and genders, its single-center design may limit the generalizability of the findings. The reliance on self-reported questionnaires could introduce bias. Additionally, the subjective assessment of treatment difficulty and the lack of consideration for co-morbidities are further limitations. Future studies should consider a larger sample size with diverse clinical settings and correlate the OHRQoL by comparing the technical quality of RCT to address these limitations.

## 5. Conclusions

Dental students are well informed about most aspects of RCT and face difficulties in locating the orifices in middle-aged patients and communicating with female patients. Physical disability and psychological discomfort were the most affected domains, and handicap was the least affected in the OHIP-14. The OHRQoL in patients with low socioeconomic status was high, and they reported greater satisfaction; however, no differences were observed between age and gender. However, the study’s findings should be interpreted considering the potential impact of recall bias among certain respondents, which was beyond the retrospective nature of this cross-sectional study.

## Figures and Tables

**Table 1:** Challenges faced by dental students during RCT: a comparison between patients’ age and gender.

Difficulties	Division	Young (%)	Old (%)	*p*-Value	Male (%)	Female (%)	*p*-Value
Communication	Easy	33 (44)	29 (38.7)	0.533 ns	49 (65.3)	31 (41.3)	0.009 *
	Average	36 (48)	36 (48)		25 (33.3)	40 (53.3)	
	Difficult	06 (8)	10 (13.3)		01 (1.3)	04 (5.3)	
Diagnosis	Easy	29 (38.7)	23 (30.7)	0.462 ns	40 (53.3)	35 (46.7)	0.113 ns
	Average	38 (50.7)	40 (53.3)		35 (46.7)	36 (48)	
	Difficult	08 (10.6)	12 (16)		0 (0)	04 (5.3)	
Rubber dam application	Easy	19 (25.3)	32 (42.7)	0.077 ns	42 (56)	36 (48)	0.389 ns
	Average	36 (48)	29 (38.7)		31 (41.3)	34 (45.3)	
	Difficult	20 (26.7)	14 (18.6)		02 (2.7)	05 (6.7)	
Access cavity preparation	Easy	26 (34.7)	25 (33.3)	0.905 ns	35 (46.7)	31 (41.3)	0.674 ns
	Average	38 (50.7)	37 (49.3)		33 (44)	34 (45.3)	
	Difficult	11 (14.7)	13 (17.4)		07 (9.3)	10 (13.4)	
Canal localization	Easy	36 (48)	18 (24)	<0.001 *	34 (45.3)	35 (46.7)	0.899 ns
	Average	32 (42.7)	30 (40)		38 (50.7)	36 (48)	
	Difficult	07 (9.3)	27 (36)		03 (4)	04 (5.3)	
Working length establishment	Easy	27 (36)	25 (33.3)	0.468 ns	36 (48)	31 (41.3)	0.328 ns
	Average	38 (50.7)	44 (58.7)		38 (50.7)	40 (53.4)	
	Difficult	10 (13.3)	06 (8)		01 (1.3)	04 (5.3)	
Instrumentation	Easy	26 (34.7)	21 (28)	0.4824 ns	37 (49.3)	31 (41.3)	0.101 ns
	Average	38 (50.7)	38 (50.7)		38 (50.7)	40 (53.4)	
	Difficult	11 (14.7)	16 (21.3)		0 (0)	04 (5.3)	
Obturation	Easy	23 (30.7)	24 (32)	0.944 ns	39 (52)	33 (44)	0.558 ns
	Average	41 (54.7)	39 (52)		35 (46.7)	40 (53.3)	
	Difficult	11 (14.7)	12 (16)		01 (1.3)	02 (2.7)	

* Statistically significant difference with *p*-value < 0.05. ns, nonsignificant.

**Table 2:** Distribution of responses (%) according to domains and individual items on the OHIP-14 scale (n = 585).

Dimension	Item	Never	Hardly Ever	Occasionally	Fairly Often	Very Often	Mean and SD	OHIP-14 Score	
								0; n (%)	>0; n (%)
Functional limitation	Difficulty speaking	432 (73.8)	107 (18.3)	24 (4.1)	22 (3.8)	0 (0)	0.535 ± 1.035	941 (80.42)	229 (19.58)
	Deterioration in the sense of taste	509 (87.0)	60 (10.3)	16 (2.7)	0 (0)	0 (0)			
Physical pain	Painful burning sensation in the mouth	485 (82.9)	77 (13.2)	15 (2.6)	8 (1.4)	0 (0)	0.662 ± 1.156	919 (78.55)	251 (21.45)
	Discomfort when eating	434 (74.2)	76 (13.0)	45 (7.7)	30 (5.1)	0 (0)			
Psychological discomfort	Lack of self-confidence	495 (84.6)	83 (14.2)	0 (0)	7 (1.2)	0 (0)	0.509 ± 0.914	915 (78.2)	255 (21.8)
	Feeling nervous	420 (71.8)	143 (24.4)	15 (2.6)	7 (1.2)	0 (0)			
Physical disability	Dissatisfaction with your diet	445 (76.1)	117 (20.0)	8 (1.4)	15 (2.6)	0 (0)	0.692 ± 1.191	864 (73.84)	306 (26.16)
	Inability to complete meals	419 (71.6)	120 (20.5)	31 (5.3)	15 (2.6)	0 (0)			
Psychological difficulty	Difficulty relaxing	395 (67.5)	122 (20.9)	53 (9.0)	15 (2.6)	0 (0)	0.621 ± 0.949	897 (76.67)	273 (23.33)
	Embarrassment	502 (85.8)	76 (13.0)	7 (1.2)	0 (0)	0 (0)			
Social disability	Irritability	447 (76.4)	108 (18.5)	23 (3.9)	7 (1.2)	0 (0)	0.571 ± 1.045	909 (77.69)	261 (22.31)
	Difficulty in functional performance	462 (79)	101 (17.3)	8 (1.4)	14 (2.4)	0 (0)			
Handicap	Inability to live	500 (85.5)	71 (12.1)	7 (1.2)	7 (1.2)	0 (0)	0.321 ± 0.872	1017 (86.92)	153 (13.08)
	Dissatisfaction with lifestyle	517 (88.4)	61 (10.4)	0 (0)	7 (1.2)	0 (0)			
Total	-	-	-	-	-	-	3.91 ± 5.523	78.9%	21.1%

The 5-point Likert scale was as follows: never = 0, hardly ever = 1, occasionally = 2, fairly often = 3, very often = 4.

**Table 3:** Relationship between patient factors and their association with RCT, comparisons by age and gender.

Factors	Variable	n (%) 585 (100)	Gender			Age		
			Males (%) 366 (62.56)	Females (%) 219 (37.44)	*p*-Value	Young (%) 385 (65.81)	Middle-Aged (%) 200 (34.19)	*p*-Value
Tooth type	Anterior	70 (11.96)	42 (11.47)	28 (12.78)	0.605 ns	48 (12.46)	22 (11)	0.14 ns
	Premolar	118 (20.17)	69 (18.85)	49 (22.37)		73 (18.96)	45 (22.5)	
	Molar	126 (21.53)	76 (20.76)	50 (22.83)		83 (21.56)	43 (21.5)	
	Anterior/Premolars	99 (16.92)	65 (17.76)	34 (15.52)		67 (17.40)	32 (16)	
	Anterior/Molars	68 (11.62)	48 (13.11)	20 (9.13)		37 (9.61)	31 (15.5)	
	Premolars/Molars	104 (17.77)	66 (18.03)	38 (17.35)		77 (20)	27 (13.5)	
Preoperative pain	Yes	390 (66.66)	259 (70.76)	131 (59.81)	0.007 *	280 (72.73)	110 (55)	<0.0001 *
	No	195 (33.33)	107 (29.24)	88 (40.19)		105 (27.27)	90 (45)	
Postoperative pain	Yes	211 (36.06)	149 (40.71)	62 (28.31)	0.003 *	146 (37.92)	65 (32.5)	0.195 ns
	No	374 (63.93)	217 (59.29)	157 (71.69)		239 (62.08)	135 (67.5)	
Socioeconomic status	High income	79 (13.50)	46 (12.57)	33 (15.07)	0.47 ns	53 (13.77)	26 (13)	0.946 ns
	Middle income	451 (77.09)	282 (77.04)	169 (77.17)		296 (76.88)	155 (77.5)	
	Low income	55 (9.40)	38 (10.39)	17 (7.76)		36 (9.35)	19 (9.5)	

* Statistically significant difference with *p*-value < 0.05. ns, nonsignificant.

**Table 4:** The mean ± standard deviation of OHRQoL for the seven domains and the total OHIP score according to patient variables.

	Variables	Functional Limitation	Physical Pain	Psychological Discomfort	Physical Disability	Psychological Difficulty	Social Disability	Handicap	Total OHIP Score
Age	Young adults	0.462 ± 0.932	0.649 ± 1.115	0.486 ± 0.905	0.686 ± 1.167	0.621 ± 0.947	0.571 ± 1.073	0.325 ± 0.893	3.8 ± 5.43
	Middle-aged adults	0.675 ± 1.199	0.685 ± 1.234	0.555 ± 0.933	0.705 ± 1.239	0.62 ± 0.954	0.57 ± 0.99	0.315 ± 0.83	3.8 ± 5.43
	*p*-value	0.018 *	0.724 ns	0.385 ns	0.853 ns	0.0.992 ns	0.987 ns	0.899 ns	0.500 ns
Sex	Male	0.574 ± 1.09	0.705 ± 1.207	0.516 ± 0.927	0.686 ± 1.226	0.623 ± 0.948	0.587 ± 1.071	0.328 ± 0.902	4.019 ± 5.735
	Female	0.47 ± 0.935	0.589 ± 1.064	0.498 ± 0.895	0.703 ± 1.133	0.616 ± 0.952	0.543 ± 1.001	0.311 ± 0.821	3.731 ± 5.158
	*p*-value	0.242 ns	0.241 ns	0.811 ns	0.863 ns	0.936 ns	0.622 ns	0.816 ns	0.541 ns
Pain before RCT	Yes	0.533 ± 1.01	0.695 ± 1.226	0.51 ± 0.869	0.7 ± 1.244	0.605 ± 0.947	0.546 ± 1.072	0.328 ± 0.949	3.918 ± 5.635
	No	0.538 ± 1.085	0.595 ± 1.003	0.508 ± 1.002	0.677 ± 1.081	0.651 ± 0.953	0.621 ± 0.989	0.308 ± 0.694	3.897 ± 5.306
	*p*-value	0.955 ns	0.324 ns	0.975 ns	0.825 ns	0.580 ns	0.417 ns	0.789 ns	0.966 ns
Pain after RCT	Yes	0.493 ± 0.953	0.588 ± 1.128	0.498 ± 0.886	0.654 ± 1.249	0.536 ± 0.962	0.507 ± 1.034	0.327 ± 0.906	3.602 ± 5.511
	No	0.559 ± 1.079	0.703 ± 1.172	0.516 ± 0.931	0.714 ± 1.158	0.668 ± 0.939	0.607 ± 1.05	0.318 ± 0.853	4.086 ± 5.53
	*p*-value	0.460 ns	0.265 ns	0.815 ns	0.560 ns	0.104 ns	0.265 ns	0.906 ns	0.310 ns
Socioeconomic status	High income	0.1899 ± 0.39471	0.6582 ± 1.0113	1 ± 1.79743	0.8481 ± 1.59397	0.6582 ± 1.22878	0.5443 ± 1.43042	0.2658 ± 0.85798	4.1646 ± 7.12603
	Middle income	0.6608 ± 1.13636	0.6763 ± 1.22269	0.4678 ± 0.67046	0.7339 ± 1.15861	0.6563 ± 0.93063	0.6275 ± 1.01041	0.3525 ± 0.91523	4.1752 ± 5.43633
	Low Income	0	0.5455 ± 0.71539	0.1455 ± 0.35581	0.1273 ± 0.33635	0.2727 ± 0.44947	0.1455 ± 0.35581	0.1455 ± 0.35581	1.3818 ± 1.75848
	*p*-value	<0.001 *	0.731 ns	<0.001 *	<0.001 *	0.017 *	0.005 *	0.208 ns	0.002 *
Tooth Type	Anterior	0.357 ± 0.835	0.357 ± 0.703	0.414 ± 0.648	0.471 ± 0.793	0.514 ± 0.775	0.386 ± 0.621	0.243 ± 0.55	2.743 ± 3.674
	Premolar	0.627 ± 1.061	0.839 ± 1.377	0.559 ± 0.711	0.949 ± 1.395	0.78 ± 1.005	0.653 ± 1.033	0.424 ± 0.973	4.831 ± 5.771
	Molar	0.563 ± 1.121	0.778 ± 1.193	0.548 ± 1.078	0.738 ± 1.221	0.659 ± 1.013	0.69 ± 1.223	0.373 ± 0.994	4.349 ± 6.052
	Anterior/Premolar	0.444 ± 0.939	0.545 ± 0.895	0.455 ± 0.76	0.475 ± 0.849	0.525 ± 0.825	0.444 ± 0.906	0.303 ± 0.801	3.192 ± 4.688
	Anterior/Molar	0.603 ± 1.067	0.515 ± 0.889	0.426 ± 0.919	0.544 ± 1.028	0.441 ± 0.853	0.588 ± 1.068	0.279 ± 0.895	3.397 ± 5.417
	Premolar/Molar	0.558 ± 1.087	0.731 ± 1.395	0.577 ± 1.163	0.798 ± 1.43	0.673 ± 1.056	0.567 ± 1.147	0.24 ± 0.818	4.144 ± 6.228
	*p*-value	0.523 ns	0.044 *	0.73 ns	0.021 *	0.148 ns	0.303 ns	0.589 ns	0.085 ns

* Statistically significant difference with *p*-value < 0.05; ns, nonsignificant; OHIP, Oral Health Impact Profile.

## Data Availability

The original contributions presented in this study are included in the article. Further inquiries can be directed to the corresponding authors.

## References

[1] Huang D., Wang X., Liang J., Ling J., Bian Z., Yu Q., Hou B., Chen X., Li J., Ye L. (2024). Expert consensus on difficulty assessment of endodontic therapy. Int. J. Oral. Sci..

[2] Alshammari F.R., Alamri H., Aljohani M., Sabbah W., O’Malley L., Glenny A.M. (2021). Dental caries in Saudi Arabia: A systematic review. J. Taibah. Univ. Med. Sci..

[3] Alqahtani A.S., Alqhtani N.R., Gufran K., Aljulayfi I.S., Alateek A.M., Alotni S.I., Aljarad A.J., Alhamdi A.A., Alotaibi Y.K. (2022). Analysis of Trends in Demographic Distribution of Dental Workforce in the Kingdom of Saudi Arabia. J. Healthc. Eng..

[4] Shah P.K., Chong B.S. (2018). A web-based endodontic case difficulty assessment tool. Clin. Oral Investig..

[5] Mirza M.B. (2015). Difficulties Encountered during Transition from Preclinical to Clinical Endodontics among Salman bin Abdul Aziz University Dental Students. J. Int. Oral. Health.

[6] Salam A.A. (2023). Ageing in Saudi Arabia: New dimensions and intervention strategies. Sci. Rep..

[7] Shakiba B., Hamedy R., Pak J.G., Barbizam J.V., Ogawa R., White S.N. (2017). Influence of increased patient age on longitudinal outcomes of root canal treatment: A systematic review. Gerodontology..

[8] Zilinskaite-Petrauskiene I., Haug S.R. (2021). A comparison of endodontic treatment factors, operator difficulties, and perceived oral health-related quality of life between elderly and young patients. J. Endod..

[9] Solomonov M., Kim H.C., Hadad A., Levy D.H., Ben Itzhak J., Levinson O., Azizi H. (2020). Age-dependent root canal instrumentation techniques: A comprehensive narrative review. Restor. Dent. Endod..

[10] Wong Y.J. (2017). Root canal treatment outcomes not affected by increasing age of patient. Evid. Based Dent..

[11] Wigsten E., Kvist T., Jonasson P., EndoReCo, Davidson T. (2020). Comparing Quality of Life of Patients Undergoing Root Canal Treatment or Tooth Extraction. J. Endod..

[12] Johnsen I., Bårdsen A., Haug S.R. (2023). Impact of Case Difficulty, Endodontic Mishaps, and Instrumentation Method on Endodontic Treatment Outcome and Quality of Life: A Four-Year Follow-up Study. J. Endod..

[13] Walter M.H., Woronuk J.I., Tan H.K., Lenz U., Koch R., Boening K.W., Pinchbeck Y.J. (2007). Oral health related quality of life and its association with sociodemographic and clinical findings in 3 northern outreach clinics. J. Can. Dent. Assoc..

[14] Hamasha A.A., Hatiwsh A. (2013). Quality of life and satisfaction of patients after nonsurgical primary root canal treatment provided by undergraduate students, graduate students and endodontic specialists. Int. Endod. J..

[15] Montero J., Lorenzo B., Barrios R., Albaladejo A., Mirón Canelo J.A., López-Valverde A. (2015). Patient-centered Outcomes of Root Canal Treatment: A Cohort Follow-up Study. J. Endod..

[16] Doğramaci E.J., Rossi-Fedele G. (2023). Patient-related outcomes and Oral Health-Related Quality of Life in endodontics. Int. Endod. J..

[17] Neelakantan P., Liu P., Dummer P.M., McGrath C. (2020). Oral health–related quality of life (OHRQoL) before and after endodontic treatment: A systematic review. Clin. Oral Investig..

[18] Seijo M.O., Ferreira E.F., Ribeiro Sobrinho A.P., Paiva S.M., Martins R.C. (2013). Learning experience in endodontics: Brazilian students’ perceptions. J. Dent. Educ..

[19] Kaplan T., Sezgin G.P., SönmezKaplan S. (2020). Dental students’ perception of difficulties concerning root canal therapy: A survey study. Saudi Endod. J..

[20] Mirza M.B., Gufran K., Alhabib O., Alzahrani F., Abuelqomsan M.S., Karobari M.I., Alnajei A., Afroz M.M., Akram S.M., Heboyan A. (2022). CBCT based study to analyze and classify root canal morphology of maxillary molars—A retrospective study. Eur. Rev. Med. Pharmacol. Sci..

[21] Braga T., Robb N., Love R.M., Amaral R.R., Rodrigues V.P., de Camargo J.M.P., Duarte M.A.H. (2021). The impact of the use of magnifying dental loupes on the performance of undergraduate dental students undertaking simulated dental procedures. J. Dent. Educ..

[22] Ab Ghani S.M., Mohd Khairuddin P.N.A., Lim T.W., Md Sabri B.A., Abdul Hamid N.F., Baharuddin I.H., Schonwetter D. (2024). Evaluation of dental students’ clinical communication skills from three perspective approaches: A cross-sectional study. Eur. J. Dent. Educ..

[23] Yuan S., Freeman R., Hill K., Newton T., Humphris G. (2020). Communication, Trust and Dental Anxiety: A Person-Centered Approach for Dental Attendance Behaviours. Dent. J..

[24] Rindlisbacher F., Davis J.M., Ramseier C.A. (2017). Dental students’ self-perceived communication skills for patient motivation. Eur. J. Dent. Educ..

[25] Alvarez S., Schultz J.H. (2018). A communication-focused curriculum for dental students–an experiential training approach. BMC Med. Educ..

[26] Swami V., McClelland A., Bedi R., Furnham A. (2011). The influence of practitioner nationality, experience, and sex in shaping patient preferences for dentists. Int. Dent. J..

[27] Yong A.J., Mohamad N., Saddki N., Ahmad W.M.A.W., Alam M.K. (2021). Patient satisfaction towards dentist-patient interaction among patients attending outpatient dental clinic hospital Universiti Sains Malaysia. Pesqui. Bras. Odontopediatria Clín. Integr..

[28] Smith M.K., Dundes L. (2008). The implications of gender stereotypes for the dentist-patient relationship. J. Dent. Educ..

[29] Santos-Puerta N., Peñacoba-Puente C. (2022). Pain and Avoidance during and after Endodontic Therapy: The Role of Pain Anticipation and Self-Efficacy. Int. J. Environ. Res. Public Health.

[30] Umanah A., Osagbemiro B., Arigbede A. (2012). Pattern of demand for endodontic treatment by adult patients in port-harcourt, South-South Nigeria. J. West. Afr. Coll. Surg..

[31] Hamedy R., Shakiba B., Fayazi S., Pak J.G., White S.N. Patient-centered endodontic outcomes: A narrative review. Iran. Endod. J..

[32] Franciscatto G.J., Brennan D.S., Gomes M.S., Rossi-Fedele G. (2020). Association between pulp and periapical conditions and dental emergency visits involving pain relief: Epidemiological profile and risk indicators in private practice in Australia. Int. Endod. J..

[33] Huang S.M., Huang J.Y., Yu H.C., Su N.Y., Chang Y.C. (2019). Trends, demographics, and conditions of emergency dental visits in Taiwan 1997–2013: A nationwide population—Based retrospective study. J. Formos. Med. Assoc..

[34] Albuquerque M.T.P., Abreu L.C., Martim L., Munchow E.A., Nagata J.Y. (2021). Tooth and patient-Related Conditions May Influence Root Canal Treatment Indication. Int. J. Dent..

[35] Wigsten E., Jonasson P., Kvist T. (2019). Indications for root canal treatment in a Swedish county dental service: Patient- and tooth-specific characteristics. Int. Endod. J..

[36] Pasqualini D., Corbella S., Alovisi M., Taschieri S., Del Fabbro M., Migliaretti G., Carpegna G.C., Scotti N., Berutti E. (2016). Postoperative quality of life following single-visit root canal treatment performed by rotary or reciprocating instrumentation: A randomized clinical trial. Int. Endod. J..

[37] Knorst J.K., Sfreddo C.S., de F Meira G., Zanatta F.B., Vettore M.V., Ardenghi T.M. (2021). Socioeconomic status and oral health-related quality of life: A systematic review and meta-analysis. Community Dent. Oral. Epidemiol..

[38] Liu P., McGrath C., Cheung G.S. (2012). Quality of life and psychological well-being among endodontic patients: A case-control study. Aust. Dent. J..

[39] Arifin F.A., Matsuda Y., Natsir N., Kanno T. (2024). Comparison of oral health-related quality of life among endodontic patients with irreversible pulpitis and pulp necrosis using the oral health-related endodontic patient’s quality of life scale. Odontology.

[40] Chung S.H., Chang J. (2021). Impact of endodontic case difficulty on operating time of single visit nonsurgical endodontic treatment under general anesthesia. BMC Oral Health.

[41] Hajek A., König H.-H., Kretzler B., Zwar L., Lieske B., Seedorf U., Walther C., Aarabi G. (2022). Does Oral Health-Related Quality of Life Differ by Income Group? Findings from a Nationally Representative Survey. Int. J. Environ. Res. Public Health.

[42] Amorim L.P., Senna M.I.B., Alencar G.P., Rodrigues L.G., de Paula J.S., Ferreira R.C. (2019). User satisfaction with public oral health services in the Brazilian Unified Health System. BMC Oral Health.

[43] Macarevich A., Pilotto L.M., Hilgert J.B., Celeste R.K. (2018). User satisfaction with public and private dental services for different age groups in Brazil. Cadernos de saude publica..

[44] Dugas N.N., Lawrence H.P., Teplitsky P., Friedman S. (2002). Quality of life and satisfaction outcomes of endodontic treatment. J. Endod..

[45] Chandraweera L., Goh K., Lai-Tong J., Newby J., Abbott P. (2019). A survey of patients’ perceptions about, and their experiences of, root canal treatment. Aust. Endod. J..

[46] Zakershahrak M., Chrisopoulos S., Luzzi L., Jamieson L., Brennan D. (2023). Income and Oral and General Health Related Quality of Life: The Modifying Effect of Sense of Coherence, Findings of a Cross Sectional Study. Appl. Res. Qual. Life.

[47] Vena D.A., Collie D., Wu H., Gibbs J.L., Broder H.L., Curro F.A., Thompson V.P., Craig R.G., PEARL Network Group (2014). Prevalence of persistent pain 3-5 years post primary root canal therapy and its impact on oral health-related quality of life: PEARL networks findings. J Endod..

[48] Sanz E., Azabal M., Arias A. (2022). Quality of Life and Satisfaction of Patients Two Years after Endodontic and Dental Implant Treatments Performed by Experienced Practitioners. J. Dent..

